# Development of Microsatellite Markers Based on Transcriptome Sequencing and Evaluation of Genetic Diversity in Swimming Crab (*Portunus trituberculatus*)

**DOI:** 10.3389/fgene.2022.932173

**Published:** 2022-07-18

**Authors:** Baohua Duan, Shumei Mu, Yueqiang Guan, Weibiao Liu, Tongxu Kang, Yana Cheng, Zejian Li, Yang Tian, Xianjiang Kang

**Affiliations:** ^1^ College of Life Sciences, Hebei University, Baoding, China; ^2^ Bureau of Agricultural and Rural Affairs of Huanghua City, Huanghua, China; ^3^ Hebei Fishery Technology Extension Station, Shijiazhuang, China; ^4^ Institute of Life Science and Green Development, Hebei University, Baoding, China; ^5^ Hebei Innovation Center for Bioengineering and Biotechnology, Hebei University, Baoding, China

**Keywords:** *Portunus trituberculatus*, transcriptome, SSR, genetic diversity, Bohai Sea

## Abstract

*P. trituberculatus* is an economically important mariculture species in China. Evaluating its genetic diversity and population structure can contribute to the exploration of germplasm resources and promote sustainable aquaculture production. In this study, a total of 246,243 SSRs were generated by transcriptome sequencing of *P. trituberculatus*. Among the examined 254,746 unigenes, 66,331 had more than one SSR. Among the different SSR motif types, dinucleotide repeats (110,758, 44.98%) were the most abundant. In 173 different base repeats, A/T (96.86%), AC/GT (51.46%), and ACC/GGT (26.20%) were dominant in mono-, di-, and trinucleotide, respectively. GO annotations showed 87,079 unigenes in 57 GO terms. Cellular process, cell, and binding were the most abundant terms in biological process, cellular component, and molecular function categories separately. A total of 34,406 annotated unigenes were classified into 26 functional categories according to the functional annotation analysis of KOG, of which “general function prediction only” was the biggest category (6,028 unigenes, 17.52%). KEGG pathway annotations revealed the clustering of 34,715 unigenes into 32 different pathways. Nineteen SSRs were identified as polymorphic and, thus, used to assess the genetic diversity and structure of 240 *P. trituberculatus* individuals from four populations in the Bohai Sea. Genetic parameter analysis showed a similar level of genetic diversity within wild populations, and the cultured population indicated a reduction in genetic diversity compared with wild populations. The pairwise *F_ST_* values were between 0.001 and 0.04 with an average of 0.0205 (*p* < 0.05), suggesting a low but significant level of genetic differentiation among the four populations. Structure analysis demonstrated that the four populations were classified into two groups including the cultured group and other populations. The phylogenetic tree and PCA revealed that a vast number of samples were clustered together and that cultivated individuals were distributed more centrally than wild individuals. The findings contribute to the further assessment of germplasm resources and assist to provide valuable SSRs for marker-assisted breeding of *P. trituberculatus* in the future.

## Introduction

Swimming crab, *P. trituberculatus*, is an edible portunid of great commercial significance, which has been widely farmed in China. Due to its high nutritional value and rapid growth, *P. trituberculatus* has become one of the most important economic crab species in marine aquaculture (Lv et al., 2017). Indeed, *P. trituberculatus* is one of the most heavily fished brachyurans in the world with approximately 95% of the total catch occurring in China (Liu et al., 2013; Hui et al., 2018). The total catch was 424,630 tons in 2020 (Bureau of Fisheries of Ministry of Agriculture, PRC, 2021). With increasing serious problems such as mass outbreaks of disease, overfishing, and water pollution in recent years, however, the *P. trituberculatus* aquaculture and fishing industry face great pressure. Despite the improved artificial propagation and rearing techniques, the crab industry still relies on the collection of wild specimens to provide parental stock (Wang et al., 2012). It is urgent to determine the genetic diversity and population structure of *P. trituberculatus* for the exploration of germplasm resources and conservation management of this species.

Genetic diversity is the material basis necessary for populations to deal with changing environment, and it can trace the history of biological evolution and explore the evolutionary potential of existing organisms (Liu et al., 2021a). However, for the breeding of shrimp and crab, long-term artificial directional selection eventually leads to a decline in genetic diversity in the population (Wang et al., 2018a). Moreover, it is difficult for natural stocks to recover from declining genetic diversity caused by overfishing (Liu et al., 2009). Benefitting from the rapid advance of high-throughput sequencing and genotyping technologies, an increasing number of molecular markers are developed and applied to genetic analyses in aquatic species. To date, molecular markers including isozyme (Fan et al., 2009), random amplified polymorphic DNA (RAPD) (Chi et al., 2010), amplified fragments length polymorphism (AFLP) (Liu et al., 2013; Liu et al., 2014), mitochondrial DNA (Guo et al., 2012; Shan et al., 2017; Hui et al., 2018), microsatellites DNA (Lee et al., 2013; Yue et al., 2022), and single nucleotide polymorphism (SNP) (Duan et al., 2022a) were developed and used in population genetic analysis of *P. trituberculatus*. Among these markers, microsatellite DNA markers (simple sequence repeats, SSRs) have become an ideal molecular marker in population genetics research because of their co-dominant inheritance, high polymorphism, reproducibility, hyper-variable, transferability, random distribution in the genome, and ease of analysis *via* PCR (Gou et al., 2020; Pavan Kumara et al., 2020; Zhu et al., 2021; Lu et al., 2022). Such markers are often used to obtain genetic diversity coefficients, which can provide a basis for genetic protection strategies. However, the traditional methods of developing SSRs are usually time-consuming and labor-intensive because of establishing of the genomic library to get the fragmented sequence and hybridization *in situ* with probes. In recent years, the increased access and affordability of high-throughput sequencing technologies have enabled genomic and transcriptomic research on many marine species, thus leading to more rapid and accurate identification of SSR markers (Yang et al., 2018; Liu et al., 2022).

In recent years, transcriptome sequencing (RNA-seq) is widely used in the study of species genetics because of its wide dynamic range, precise, sensitivity, unbiased quantification of transcripts, and comprehensive coverage of all expressed sequences in a given tissue sample (Chakraborty et al., 2022). Now, RNA-seq is a very updated and efficient method for discovering new genes, expression pattern identification, and development of SSR markers with higher throughput and much lower cost (Li et al., 2018; Tulsania et al., 2020). SSR markers acquired from RNA-seq have intrinsic advantages over genomic SSRs because of high efficiency, strong transferability, and correlation with potential genes, as well as their applicability as anchor markers for comparative mapping and evolutionary studies (Zheng et al., 2014; Zhao et al., 2019). Transcriptomic SSRs have been extensively explored and applicated in various aquatic species, such as giant freshwater prawn (*Macrobrachium rosenbergii*) (Jung et al., 2011; Yu et al., 2019), oriental river prawn (*Macrobrachium nipponense*) (Ma et al., 2012), mud crab (*Scylla paramamosain*) (Ma et al., 2014), *Paphia textile* (Chen et al., 2016), *Penaeus monodon* (Nguyen et al., 2016), and pearl oyster (*Pinctada maxima*) (Wang et al., 2019a). Transcriptomics have also played an important role in advancing portunid aquaculture (Waiho et al., 2022). Nevertheless, the current transcriptome studies involving *P. trituberculatus* mainly focus on its nutrition (Zhou et al., 2019a; Fang et al., 2021a), development (Liu et al., 2018a; Liu et al., 2019), reproduction (Wang et al., 2018b), and sex determination (Zhang et al., 2022), while molecular research is scarce.

The present study aimed to 1) develop SSR markers with RNA-seq technology; 2) characterize the transcriptome of *P. trituberculatus*; and 3) evaluate the genetic diversity and structure among different populations using the polymorphic SSR markers from transcriptome sequencing*.* Our findings not only contribute to molecular genetic analyses but also provide valuable information for effective breeding and conservation strategies of the *P. trituberculatus* aquaculture.

## Materials and Methods

### Sample Collection and DNA Extraction

**FIGURE 1:**
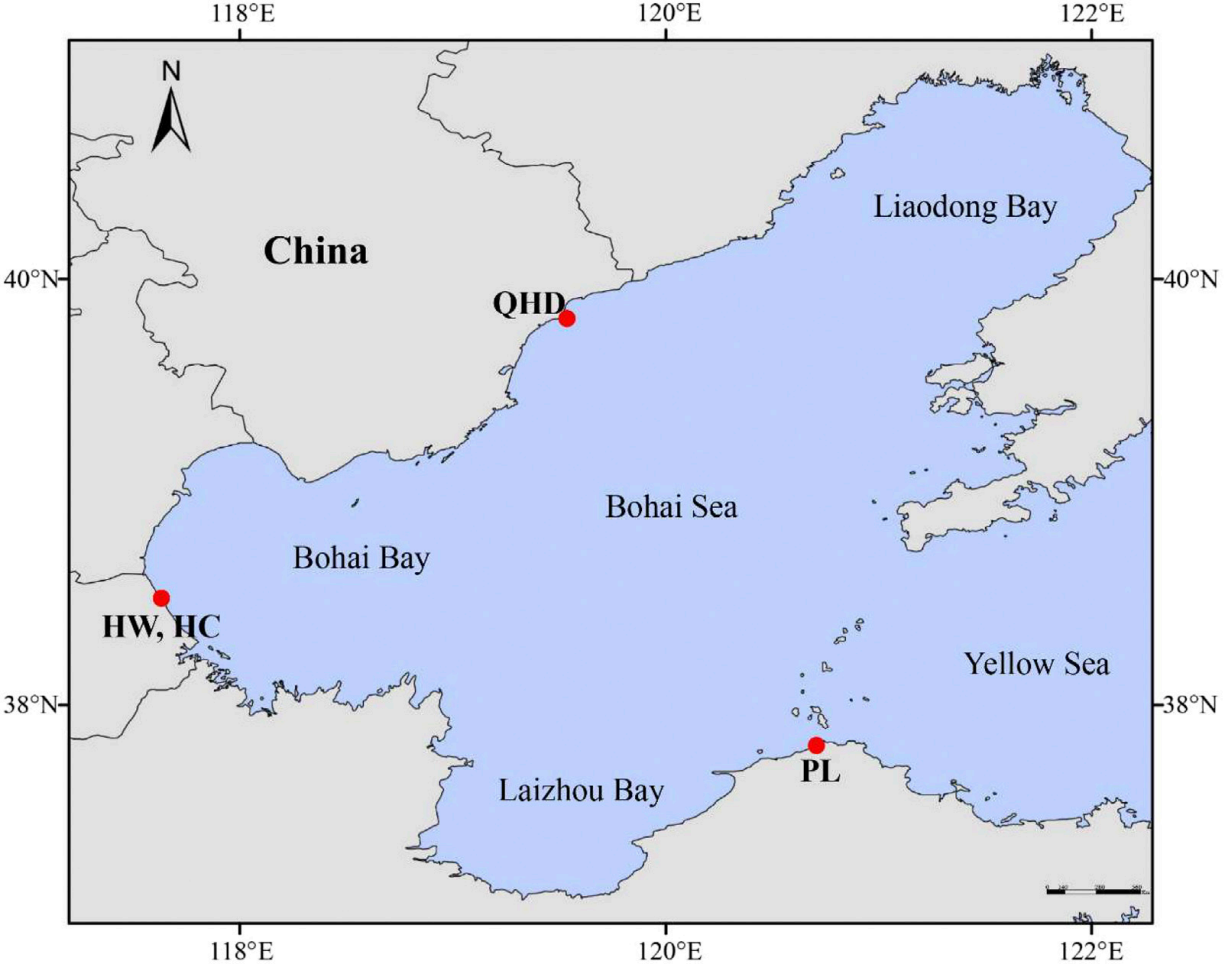
Collection map of *P. trituberculatus* samples.

A total of 240 swimming crab samples from four populations were collected (Supplementary Table S1; Figure 1), including three wild populations [Qinhuangdao (QHD), Huanghua (HW), and Penglai (PL)] from the Bohai Sea and one cultured stock (HC) from the national breeding farm of swimming crabs in Huanghua (Hebei, China) which is adjacent to the Bohai Sea. Claws of each sample were obtained and preserved in absolute alcohol and stored at −20°C until DNA extraction. Genomic DNA was isolated from the claw muscle using the TIANamp Marine animal DNA extraction kit (TIANGEN, Beijing, China) following the manufacturer’s protocols. The quality and concentration of the extracted DNA were determined using the NanoDrop ND-1000 spectrophotometer (Thermo Scientific, Wilmington, DE, United States) and 1% agarose electrophoresis gel, and then diluted to 100 ng/μl and stored at −20°C for polymerase chain reaction (PCR) amplification.

### RNA Extraction, Library Preparation, and Transcriptome Sequencing

A total of 18 individuals *P. trituberculatus* (nine wild females and nine cultured females) were collected from the national breeding farm of swimming crabs in Huanghua, China. The crabs were all anesthetized on ice and dissected to collect muscle and ovaries samples. All of the samples were rapidly flash-frozen in liquid nitrogen and stored at −80°C for RNA extraction. An equal amount of either muscle tissue or ovaries tissue was dissected from each of three individuals and pooled into one mixed sample. A total of 12 mixed samples were produced, including 6 mixed muscle samples (3 wild and 3 farmed) and 6 mixed ovarian samples (3 wild and 3 farmed), each containing three biological replicates. Total RNA was extracted from each mixed sample using the TRIzol reagent (Invitrogen, Carlsbad, CA, United States) following the manufacturer’s protocol. RNA degradation and contamination were monitored on 1% agarose gels, and RNA purity was checked using the NanoPhotometer^®^ spectrophotometer (IMPLEN, CA, United States). RNA concentration and integrity were measured using the Qubit^®^ RNA Assay Kit in Qubit^®^ 2.0 Flurometer (Life Technologies, CA, United States) and the RNA Nano 6000 Assay Kit of the Agilent Bioanalyzer 2100 system (Agilent Technologies, CA, United States), respectively.

A total amount of 1.5 μg RNA from each sample was used as input material for the RNA sample preparations. Sequencing libraries were generated using the NEBNext^®^ Ultra™ RNA Library Prep Kit for Illumina^®^ (NEB, United States) following the manufacturer’s recommendations and index codes were added to attribute sequences to each sample. Briefly, mRNA was purified from total RNA using poly-T oligo-attached magnetic beads. Fragmentation was carried out using divalent cations under elevated temperature in NEBNext First Strand Synthesis Reaction Buffer (5X). First-strand cDNA was synthesized using random hexamer primer and M-MuLV Reverse Transcriptase (RNase H^−^). Second strand cDNA synthesis was subsequently performed using DNA Polymerase I and RNase H. Remaining overhangs were converted into blunt ends *via* exonuclease/polymerase activities. After adenylation of 3′ ends of DNA fragments, NEBNext Adaptor with hairpin loop structure was ligated to prepare for hybridization. In order to select cDNA fragments of preferentially 250–300 bp in length, the library fragments were purified with AMPure XP system (Beckman Coulter, Beverly, United States). Then 3 μl USER Enzyme (NEB, United States) was used with size-selected, adaptor-ligated cDNA at 37°C for 15 min followed by 5 min at 95°C before PCR. Subsequently, PCR was performed with Phusion High-Fidelity DNA polymerase, Universal PCR primers, and Index (X) Primer. Then PCR products were purified (AMPure XP system) and library quality was assessed on the Agilent Bioanalyzer 2100 system. At last, the library preparations were sequenced on an Illumina HiSeq 4000 platform and paired-end reads were generated at Novogene Corporation (Tianjin, China).

### Quality Control, Transcriptome Assembly, and Gene Function Annotation

Raw data (raw reads) of fastq format were firstly processed through in-house perl scripts. In this step, the clean data (clean reads) were obtained by removing reads containing adapter, reads containing ploy-N, and low-quality reads (quality score < 20) from raw data. At the same time, Q20, Q30, GC-content, and sequence duplication levels of the clean data were calculated. All the downstream analyses were based on clean data with high quality. Transcriptome assembly of the high-quality clean reads was accomplished using Trinity software with default settings (Grabherr et al., 2011). In order to annotate the assembled unigenes, a BLASTX search with an E-value < 10^−5^ (Camacho et al., 2009) was performed against several public databases, including NCBI non-redundant protein sequences (Nr), NCBI non-redundant nucleotide sequences (Nt), Protein family (PFAM), euKaryotic Ortholog Groups (KOG), Swiss-Prot protein, KEGG Ortholog database (KO) and Gene Ontology (GO). Assigning the GO terms to the unigenes was implemented on Blast2GO software (Götz et al., 2008).

### Simple Sequence Repeats Identification and Primer Design

SSR loci were identified throughout all unigenes generated by the *P. trituberculatus* transcriptome sequencing using MISA software version 1.0 (http://pgrc.ipk-gatersleben.de/misa/misa.html). The minimum number of repeats was defined as ten for mononucleotide repeats, six for dinucleotide repeats, five for tri-, tetra-, penta-, and hexanucleotide repeats. Primer pairs for each SSR locus were designed using Primer3 (http://primer3.sourceforge.net/releases.php) according to the following criteria: 1) primer length of 18–25 bp; 2) annealing temperature (Tm) between 55°C and 62°C; 3) GC content from 40% to 60%; 4) PCR product length of 90–250 bp; 5) avoidance of primer dimers and hairpin structures. SSR primers were synthesized by General Biosystems Co., Ltd. (Anhui, China). Twelve samples were used to identify the polymorphism of the selected SSR primers through PCR amplification and 8% non-denaturing polyacrylamide gel electrophoresis with pBR322 DNA/MspI (MBI) as a standard DNA marker.

### Simple Sequence Repeat Genotyping

A total of 19 pairs of polymorphic SSR primers were identified and used for subsequent analysis (Table 1). All forward primers were labeled with the fluorescent dye, 6-carboxy-fluorescein (FAM). PCR amplification was performed in 20 μl reaction volumes containing 2 μl of template DNA, 2 μl of each primer (2.5 μmol/L each), 10 μl of 2 × Es Taq Master Mix (CWBIO, Beijing, China), and 4 μl of ddH_2_O. Amplification cycles consisted of initial denaturation (5 min at 95°C), followed by 34 cycles of denaturation (30 s at 95°C), annealing (30 s at 60°C), extension (30 s at 72°C), and further extension (10 min at 72°C). After amplification, PCR products were diluted 10 times in sterile water. The pooled sample was composed of 20 μl Hi-Di formamide and 0.2 μl GeneScan 500 ROX Size Standard. An ABI 3730XL Genetic Analyzer (Applied Biosystems, Foster City, CA, United States) was used to conduct capillary electrophoresis (CE) following the manufacturer’s instructions. Each CE sample contained 1 μl diluted PCR product and 15 μl pooled sample. Allele sizes (in base pairs) were determined with GeneMarker^®^ Fragment Analysis Software (Softgenetics LLC^®^, State College, PA, United States) on the comparison of the position of the internal size standard in each lane with the position of the peak value of each sample.

**TABLE 1:** Characteristics of 19 SSR loci for *P. trituberculatus*.

Locus	Primer sequences	Repeat motif	Anneal (°C)
PrMa01	F:CCTTGCCTCGTCAGTGTCAT	(CTG)6	60
	R:TGGCTGTAGACACCCTCCAT		
PrMa02	F:AGAGCTGACCTCGCTTTGAC	(GTG)8	60
	R:TCCAGCTCCTCCTGTCCAAT		
PrMa03	F:CTTGATTGCCTCTCGCTTGT	(TG)10	60
	R:GGGGGAGAGGGAGAGAATGT		
PrMa04	F:TCCTGGACCTTGTTCAGTCC	(TCC)10	60
	R:GCAATCCCACACACACTCCT		
PrMa05	F:GCGTTGCGTGTACTGAAAGT	(TG)31	60
	R:GCGGCTCTGGTCAGGAATAC		
PrMa06	F:TCCTGCAACTTACATTCTTGGTC	(CA)15	60
	R:GTGTGCACAGGATACAGCCT		
ZL05	F:AGAATGTTGCCATGGCTGGA	(GGT)7	60
	R:ACCCTGTATCAGTGCGTTGG		
ZL06	F:CCCGCCCCTGTACATTTTCA	(TAT)10	60
	R:TGTTGGTAGGCTTGGTGGTC		
ZL08	F:GCTTCTGCTGCTGGTCCTTA	(CAAC)10	60
	R:ACCAGACATTGCTGAGCATG		
DX05	F:GTGGGCCGCCAATATCACTA	(TG)12	60
	R:AATCCACCACTTGCACCCAA		
DX07	F:CGTGCATCCGTGTGTTTGTT	(TG)10	60
	R:GCCATCTTTTCGCCGAGTTG		
DX09	F:TAGGCATGGGATGGGTGAGA	(CA)17	60
	R:CGGGAAGGAGTGTTGTTGAGT		
DX10	F:AATCACAACCCAGCCGCATA	(TG)12	60
	R:ACAACGAAGGAGAGATGCGG		
DX14	F:CCCGCTACCCCATAACTCAC	(GTG)7	60
	R:TCTTCCTCCCCACAGCCATA		
DX15	F:CGTCCCATCATCTGACAAAGG	(GAG)6	60
	R:TCCTTCACCTCTTCCTCTTTTCT		
DX16	F:GAGGCAAGCAAGTTAACCATTAG	(GT)7	60
	R:CTTCCTGGTTACCTCATCCTACC		
DX19	F:CACACTCGTTGCAGACACTACTT	(TG)11	60
	R:CTGTTACTTACTCGGTGCTTTGG		
TRAN2	F:TCACTACCACTACCGCTTTGTTT	(CAC)8	60
	R:GATGTCAGTAACGGGAGAGTGAG		
TRAN3	F:GCTGTTGTAGAAACCCATGAAAG	(GTG)7	60
	R:AGGGAGATACACGACCAACACTA		

### Genetic Diversity and Population Structure Analysis

Genetic diversity was estimated by determining the genetic parameters, such as the number of alleles (*Na*), the effective number of alleles (*Ne*), the observed heterozygosity (*H*_*O*_) and expected heterozygosity (*H*_*E*_) using POPGENE version 1.3 (Yeh et al., 1999). Based on allele frequency, the polymorphism information content (*PIC*) per locus was estimated by PIC-CALC software (Nagy et al., 2012). The null allele frequency (*F*na) for loci was calculated using GenePOP (Rousset, 2008). *p* values were calculated for determining the Hardy–Weinberg equilibrium (HWE) at each locus with POPGENE version 1.3. Genetic differentiation index (*F*_*ST*_) between populations and analysis of molecular variance (AMOVA) were calculated using GenAlEx 6.5 (Peakall and Smouse, 2012).

The phylogenetic tree was constructed using the neighbor-joining (NJ) method as implemented in MEGA7 (Kumar et al., 2016). Principal component analysis (PCA) was carried out using Canoco 4.5 to elucidate genetic relationships within and among populations. Bayesian model-based population genetic structure was inferred by STRUCTURE version 2.3.4 (Pritchard et al., 2000). The putative number of populations (K) was set from 1 to 10 with 3 replicate simulations for each K value using 100,000 MCMC (Markov Chain Monte Carlo) iterations after an initial 100,000 burn-in period. With the log probability of data (LnP(D)) and an *ad hoc* statistic ΔK based on the rate of change in LnP(D) between successive K-values, the structure output was entered into Structure Harvester (Evanno et al., 2005; Earl and Vonholdt, 2012) to determine the optimum K value.

## Results

### Transcriptome Assembly and Sequence Annotation

The transcriptome sequencing of 12 mixed samples from muscle and ovary of *P. trituberculatus* was conducted to generate RNA sequences, and the statistical data has been shown in Table 2. Illumina sequencing generated 661,922,456 raw reads. The raw reads produced in this study have been deposited in the Short Read Archive of the National Center for Biotechnology Information (NCBI) with accession numbers SUB11453401 and PRJNA836158. After stringent quality filtering, a total of 637,983,466 clean reads were obtained, accounting for 99.38% of the total raw reads. GC content ranged from 48.5% to 53.47% with an average of 50.88%, and the mean Q20 and Q30 were 96.3% and 91.07%, respectively. A total of 338,285 transcripts were identified with an average length of 879 bp (N50 length of transcript = 1,730 bp, which is defined as the shortest sequence length of 50% of total contigs and is used to evaluate the quality of assembled sequences), of which 11,0596 (32.69%) were less than 301 bp in length; 84,886 (25.09%) were 301–500 bp; 61,915 (18.3%) were 501–1,000 bp; 43,085 (12.74%) were 1,001–2,000 bp; 37,803 (11.18%) were over 2,000 bp (Supplementary Figure S1). Totaling 254,746 unigenes were assembled with an average length of 1,077 bp (N50 length of unigenes is 1,936 bp), among which 47,174 (18.52%) were less than 301 bp in length; 67,149 (26.36%) were 301–500 bp; 59,692 (23.43%) were 501–1,000 bp; 42,928 (16.85%) were 1,001–2,000 bp; 37,803 (14.84%) were over 2,000 bp (Supplementary Figure S1).

**TABLE 2:** Summary statistics for transcriptome sequencing of *P. trituberculatus*.

Category	Number
Total raw reads	661,922,456
Total clean reads	637,983,466
Clean reads proportion (%), Q20 (%), Q30 (%)	99.38, 96.3, 91.07
Total number of unigenes examined	254,746
Mean length of unigenes (bp), N50 (bp)	1,077, 1,936
GC content (%)	50.88
Total amount of transcripts	338,285
Mean length of transcripts (bp), N50 (bp)	879, 1,730
Total size of examined sequences (bp)	274,270,543
Total number of identified SSRs	246,243
Number of SSR containing sequences	132,908
Number of sequences containing more than one SSR	66,331

GO database was the largest matched database with 87,079 unigenes (34.18% of all unigenes) annotated, followed by PFAM (86,669, 34.02%), Nr (77,856, 30.56%), SwissProt (58,305, 22.88%), KO (34,715, 13.62%), KOG (34,406, 13.5%), and Nt (29,269, 11.48%) database (Supplementary Figure S2). In all, 118,572 (46.54%) unigenes were annotated in at least one database and 9,901 (3.88%) were annotated in all databases. In the Nr databases, 77,856 unigenes were annotated from 835 species. The top-hit species in similarity search against the Nr database included *Zootermopsis nevadensis* (9,854, 12.7%), *Daphnia pulex* (4,798, 6.2%), *Tribolium castaneum* (2,730, 3.5%), *Stegodyphus mimosarum* (2,399, 3.1%), *Crassostrea gigas* (2,389, 3.1%), and other (55,686, 71.5%) (Supplementary Figure S3).

**FIGURE 2:**
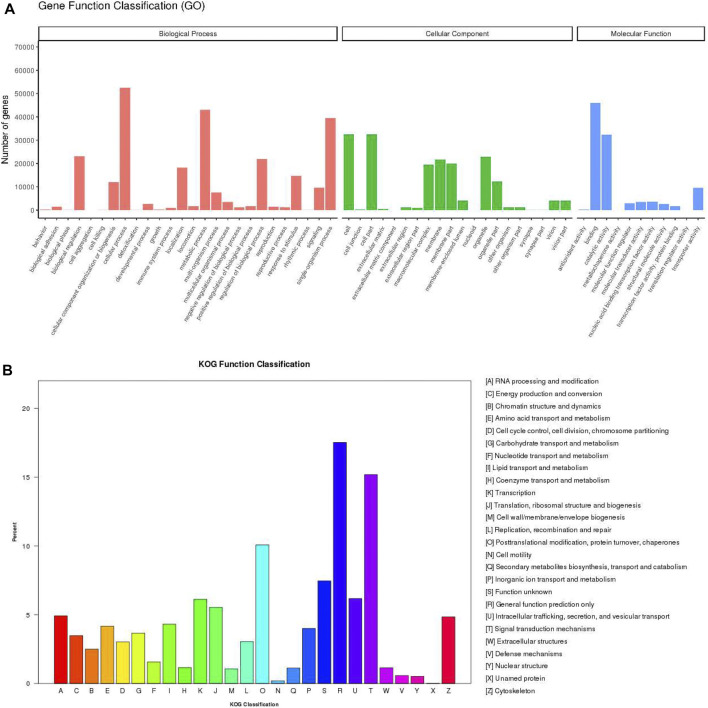
Annotation of the *P. trituberculatus* transcriptome. **(A)** GO annotation and classification of unigenes. **(B)** KOG annotation and classification of unigenes. The x-axis indicates the categories, and the y-axis indicates the number of the unigenes.

For the functional annotation and classification of the assembled unigenes, 87,079 unigenes were assigned to 57 GO terms which included three ontology categories: biological process (258,416 unigenes), cellular component (178,933), and molecular function (102,370) (Figure 2A). The main components within biological process category contained cellular process (52,412, 60.19%), metabolic process (43,020, 49.40%), and single-organism process (39,395, 45.24%). Cell (32,536, 37.36%) and cell part (32,535, 37.36%) were the most frequent proportion in cellular component category. In the molecular function category, the largest potion was binding (45,970, 52.79%), followed by catalytic activity (32,373, 37.18%). According to KOG annotations, 34,406 annotated unigenes were classified into 26 functional categories (Figure 2B). Among these categories, “general function prediction only” was the biggest category (6,028 unigenes, 17.52%), followed by “signal transduction mechanisms” (5,223, 15.18%), and “posttranslational modification, protein turnover, chaperones” (3,466, 10.02%) category.

**FIGURE 3:**
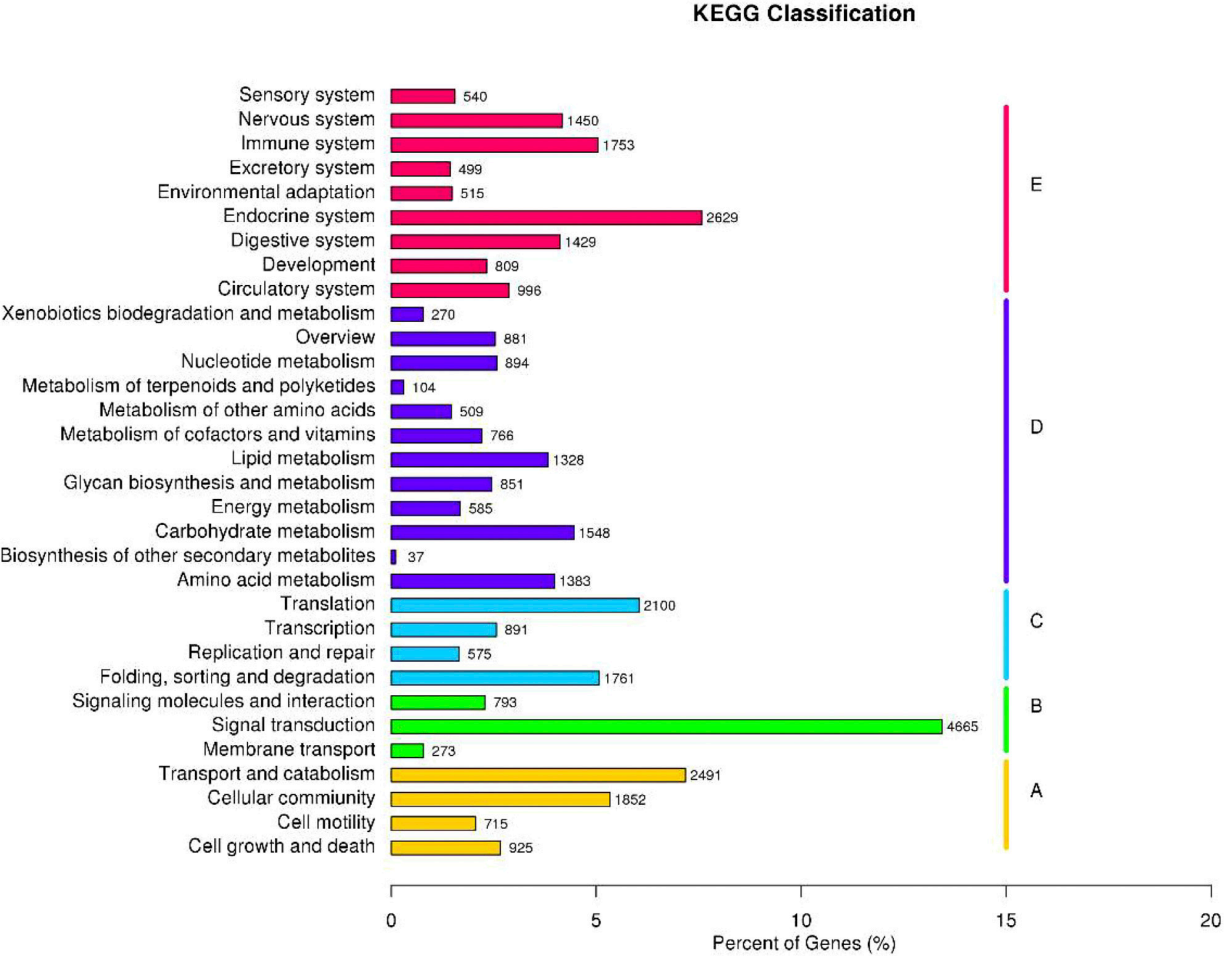
Function annotation and classification of *P. trituberculatus* unigenes in the KEGG category.

Identification of the biological pathways was performed according to the KEGG annotations, which showed the clustering of 34,715 unigenes into 32 pathways (Figure 3). Detailedly, these unigenes were categorized into five KEGG biochemical pathways: Cellular Processes (A), Environmental Information Processing (B), Genetic Information Processing (C), Metabolism (D), and Organismal Systems (E). This analysis revealed that the top five pathways included signal transduction (4,665 unigenes, 13.44%), endocrine system (2,629, 7.57%), transport and catabolism (2,491, 7.18%), translation (2,100, 6.05%), and cellular community (1,852, 5.33%).

### Characterization of Simple Sequence Repeat Markers

A total of 246,243 SSRs were identified from 132,908 SSR-containing unigenes of *P. trituberculatus*. The distribution density was one SSR per 1.11 kb, and 66,331 sequences contained more than one SSR (Table 2)*.* Dinucleotide repeats were the most abundant type (110,758, 44.98%), followed by mono- (64,679, 26.27%), tri- (61,923, 25.15%), tetra- (7,154, 2.91%), penta- (1,343, 0.55%), and hexanucleotide (386, 0.16%) (Supplementary Table S1). The number of tandem repeats of microsatellite motifs ranged from 5 to 103. Microsatellites with six tandem repeats (28371, 11.52%) were the most common, followed by five tandem repeats (25,920, 10.53%), ten tandem repeats (24,777, 10.06%), and eleven tandem repeats (24,136, 9.88%). Microsatellite motifs with > 16 tandem repeats accounted for 22.39% (55,129) (Supplementary Table S2).

Of the 246,243 SSR loci, 173 different repeat motifs were detected (Supplementary Table S3). Among the two types of mononucleotide repeats, A/T was the most abundant (62,646, 96.85%) when compared to C/G. AC/GT (56,994, 51.46%) was the dominant motif type in the dinucleotide repeat, followed by AG/CT (47,487, 42.87%), and AT/AT (6,046, 5.46%). The most abundant types in the trinucleotide were ACC/GGT (16,224, 26.20%). Moreover, a low percentage (3.62%) of tetra-, penta- and hexanucleotide repeat motifs were observed in all identified microsatellite motifs. The physical positions of these SSR markers in the unigenes were also identified that 1708, 8,644, and 5,067 SSRs were located in the coding sequence (CDS), 3′untranslated region (UTR) and 5′UTR, respectively. In CDS, trinucleotide repeats (1,279, 74.88%) were the dominant type. Most of the mono- and dinucleotide repeats (6,290, 72.77%) were located in 3′UTR, and 5′UTR contained the majority of di- and trinucleotide repeats (3,283, 64.79%) (Supplementary Figure S4).

A total of 104,424 pairs of SSR primers were designed, 150 of which were randomly selected to identify polymorphism (Supplementary Table S4). Finally, 19 pairs of SSR primers showed high polymorphism in 8% non-denaturing polyacrylamide gel electrophoresis (Table 1), and they were used for subsequent analysis in *P. trituberculatus*.

### Genetic Diversity Within Populations

All parameters of the 19 SSR loci were calculated (Table 3). The *PIC* values ranged from 0.598 (TRNA3) to 0.948 (PrMa05) with a mean of 0.802, showing that these SSRs has high polymorphism (*PIC* > 0.5) and are suitable for the evaluation of genetic diversity in *P. trituberculatus* populations. A total of 243 alleles were found with an average of 12.79 per locus. The *Ne* values ranged from 2.751 to 20.156 with a mean of 7.095. The *H*_*O*_ and *H*_*E*_ ranged from 0.467 to 0.938 (mean: 0.692) and from 0.636 to 0.95 (mean: 0.824), respectively. The three wild populations (HW, PL, and QHD) showed a similar level of genetic diversity, while the cultivated population indicated a reduction in genetic diversity compared with them due to the relatively smaller genetic parameters (Table 4). Notably, the majority of SSR loci had null alleles and presented significant deviations from HWE, and heterozygote deficiency (*H*_*O*_ < *H*_*E*_) was observed, with the exception of PrMa01, DX05, and DX19.

**TABLE 3:** Genetic diversity parameters for 19 SSR loci.

Locus	*Na*	*Ne*	*H_O_*	*H_E_*	*PIC*	*F*na	HWE
PrMa01	12	5.124	0.925	0.805	0.779	0.0000	**
PrMa02	11	7.354	0.532	0.864	0.849	0.0706	**
PrMa03	21	11.161	0.833	0.91	0.903	0.0486	**
PrMa04	12	6.672	0.808	0.85	0.834	0.0157	**
PrMa05	30	20.156	0.791	0.95	0.948	0.084	**
PrMa06	20	9.693	0.488	0.897	0.888	0.2086	**
ZL05	9	4.239	0.467	0.764	0.727	0.0991	**
ZL06	11	5.079	0.708	0.803	0.782	0.0788	**
ZL08	12	6.644	0.82	0.85	0.833	0.000	ns
DX05	10	6.363	0.871	0.843	0.824	0.000	ns
DX07	7	3.892	0.613	0.743	0.702	0.0254	**
DX09	21	12.708	0.774	0.921	0.916	0.0835	**
DX10	10	4.938	0.699	0.798	0.773	0.0525	**
DX14	8	4.052	0.525	0.753	0.714	0.0289	**
DX15	13	8.75	0.571	0.886	0.875	0.1829	**
DX16	7	4.222	0.679	0.763	0.727	0.0359	**
DX19	11	6.647	0.938	0.85	0.832	0.000	**
TRAN2	9	4.367	0.629	0.771	0.736	0.000	**
TRAN3	9	2.751	0.483	0.636	0.598	0.000	**
Mean	12.79	7.095	0.692	0.824	0.802		

*Na*, number of alleles; *Ne*, number of effective alleles; *Ho*, observed heterozygosity; *H_E_*, expected heterozygosity; *PIC*, polymorphism information content; *F*na, frequency of null alleles; HWE, Hardy–Weinberg equilibrium; **p < 0.01. ns, no deviations from HWE.

**TABLE 4:** Mean genetic parameters of four *P. trituberculatus* populations.

Population	*Na*	*Ne*	*H_O_*	*H_E_*	*PIC*
HW	11.421	5.572	0.706	0.756	0.728
PL	11.842	5.595	0.7	0.76	0.731
QHD	11.263	5.802	0.675	0.752	0.724
HC	8.737	4.507	0.688	0.716	0.679

### Population Differentiation and Variation

The populations HW and HC showed highest *F*_*ST*_ (0.040) (*p* < 0.05) whereas the lowest *F*_*ST*_ of 0.001 (*p* < 0.05) was observed between populations PL and QHD (Table 5). The mean *F*_*ST*_ was observed to be 0.0205, indicating low but significant levels of genetic differentiation among the four populations. The results of AMOVA revealed that only 2% of genetic variation was partitioned among populations while 98% of the variation was concentrated within populations (Table 6).

**TABLE 5:** Estimates of pairwise *F_ST_* values among the four *P. trituberculatus* populations.

	HW	HC	PL	QHD
HW	—			
HC	0.040*	—		
PL	0.016*	0.023*	—	
QHD	0.020*	0.023*	0.001*	—

*Significant difference (*p* < 0.05).

**TABLE 6:** Analysis of molecular variance (AMOVA) from four *P. trituberculatus* populations.

Source of variation	*df*	*SS*	*MS*	Variance component	Percentage of variation (%)	*p*-value
Among populations	3	77.042	25.681	0.149	2	0.001
Within populations	476	3408.842	14.333	7.166	98	0.001
Total	479	3485.883		7.316	100	0.001

*df*, degrees of freedom; *SS*, sum of squares; *MS*, mean square.

### Population Genetic Structure

**FIGURE 4:**
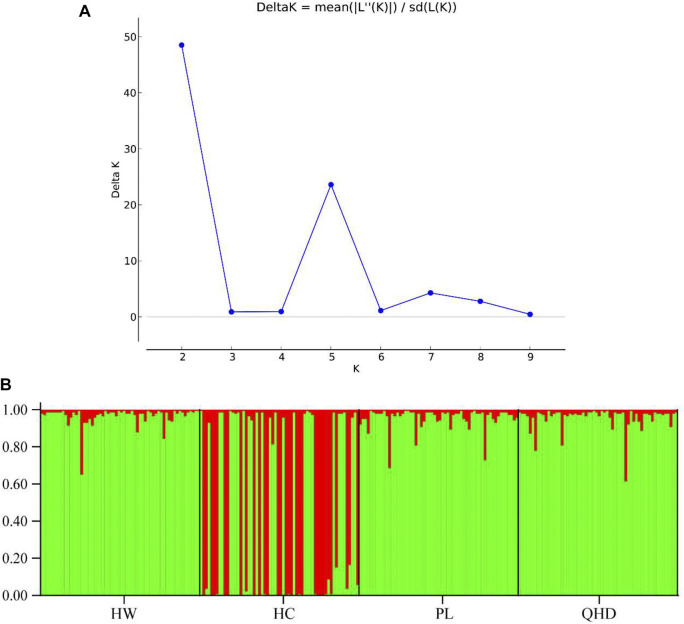
**(A)** Relationships between the number of clusters (K) and the corresponding. ΔK statistics calculated based on STRUCTURE analysis. **(B)** Population genetic structure based on the Bayesian clustering model among 240 *P. trituberculatus* samples at K = 2.

**FIGURE 5:**
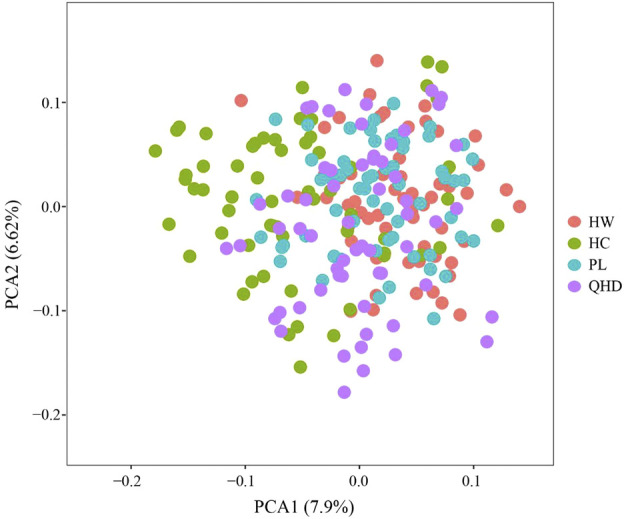
PCA analyses based on SSR data for 240 individuals from 4 *P. trituberculatus* populations.

**FIGURE 6:**
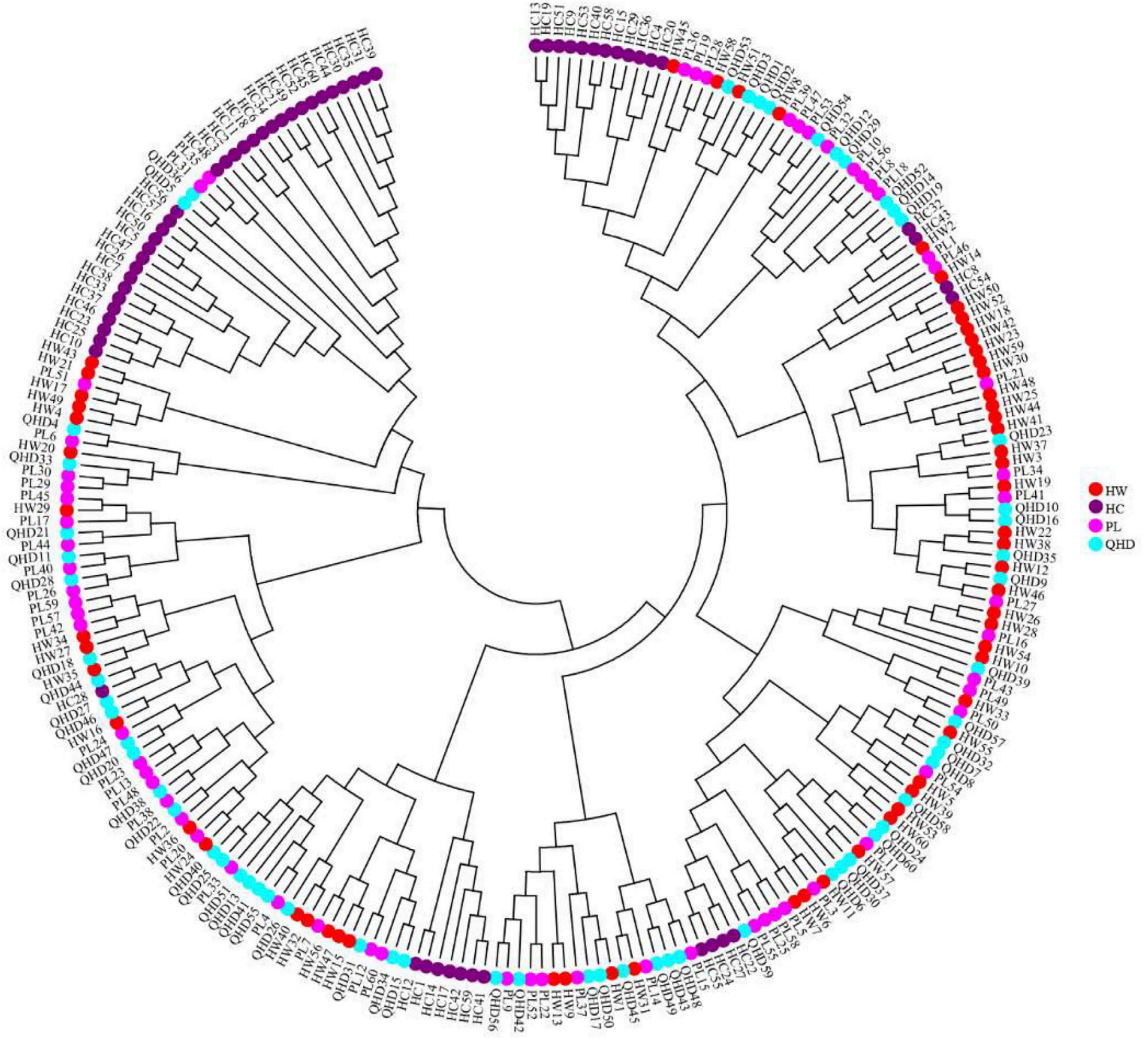
Neighbor-joining phylogenetic tree of 240 individuals of *P. trituberculatus* based on SSR data.

The genetic structural analysis of 240 *P. trituberculatus* samples was performed to infer the optimal K value with the ΔK method. When the highest ΔK value was observed, the optimal K value was 2 (Figure 4A), indicating that all individuals were clustered into two groups, including wild group (green) and the cultivated group (red) (Figure 4B). This was consistent with the population-level phylogenetic tree that the four populations were divided into 2 main clusters, in which cluster 1 contained only HC and cluster 2 contained all wild populations (Supplementary Figure S5). A certain degree of biological mixing, however, was also observed between wild and cultivated samples. The PCA revealed that the first two principal components (PCs) explained 7.9% (PC1) and 6.62% (PC2) of total variation respectively (Figure 5). The majority of samples were clustered together, and no obvious geographical patterns were observed. The cultivated individuals were mainly clustered towards the right side (positive values) of PC1. The individual-level phylogenetic tree was constructed based on NJ method, in which all individuals were clustered into two clades, and no significant clustering patterns related to geographical locations were found, but cultivated individuals were distributed more centrally than wild individuals (Figure 6).

## Discussion

### Transcriptome Sequencing

*P. trituberculatus* is a typical mariculture species with significant economic value. The Bohai Sea is an important habitat and fishing area for this species. The germplasm resources from the Bohai Sea form a vital foundation for the protection and breeding of *P. trituberculatus* in the national breeding farms of swimming crabs. Large-scale development of molecular markers and advancement of high-throughput sequencing technologies provide a solid support for germplasm resources assessment of *P. trituberculatus* in recent years (Shan et al., 2017; Liu et al., 2021b). Lv et al. (2014) identified 22,673 SSR markers of *P. trituberculatus* with transcriptome sequencing, which provided a material basis for future genetic linkage and quantitative trait loci analyses in this species. In this study, Illumina sequencing of *P. trituberculatus* was performed for the development of SSR markers, which generated new high-throughput data for transcriptomics, providing valuable information for germplasm conservation and selective breeding of *P. trituberculatus*.

A total of 246,243 SSRs were identified with the Illumina HiSeq 4000 platform, which was far higher than that of yesso scallop (*Patinopecten yessoensis*) (2,748 SSRs, Hou et al., 2011), yellow drum (*Nibea albiflora*) (12,254, Gong et al., 2016), Xingguo red carp (*Cyprinus carpio* var.*singuonensis*) (13,652, Yue et al., 2016), freshwater ornamental shrimps (*Neocaridina denticulate*) (25,355, Huang et al., 2020), and clam (*Cyclina sinensis*) (12,418, Fang et al., 2020). The possible reason is that *P. trituberculatus* has more chromosomes (2n = 106) than shellfish, fish, and other crustaceans (Liu et al., 2012), thus containing more SSR sequences. The average density of SSRs was 1/1.11 kb, which was higher than that in *P. maxima* (1/29.73 kb) (Wang et al., 2019a), sea cucumber (*Apostichopus japonicus*) (1/29.2 kb) (Du et al., 2012), mandarin fish (*Siniperca chuatsi*) (1/26.28 kb) (Sun et al., 2019), large-scale loach (*Paramisgurnus dabryanus*) (1/6.99 kb) (Li et al., 2015a), giant wrasse (*Cheilinus undulatus*) (1/5.35 kb) (Liu et al., 2020), burbot (*Lota lota*) (1/4.25 kb) (Meng et al., 2019), and the Bombay duck (*Harpadon nehereus*) (1/3 kb) (Huang et al., 2021), while lower than that of the South African abalone (*Haliotis midae*) (1/0.756 kb) (Franchini et al., 2011), tu-chung (*Eucommia ulmoides*) (1/0.73 kb) (Huang et al., 2013). The differences between SSR frequency and density may be attributed to genome structure, SSR mining tools, dataset size, and search criteria (Liu et al., 2021c). In addition, the most abundant types of SSR are dinucleotide repeats, which was consistent with the conclusions obtained by other high-throughput sequencing technologies in aquatic animals (Kong et al., 2019; Zhai et al., 2020).

In 173 different repeat motifs from the identified microsatellites in this study, A/T was the most abundant motif type higher than C/G in mononucleotide, which was congruent with the previous studies (Song et al., 2008; Fang et al., 2020; Tian et al., 2021). Ni et al. (2018) indicated that DNA recombination and replication sliding mechanisms in PCR amplification might result in high A/T content. In addition, methylated cytosine C is easily mutated into thymine T through deamination, which makes G/C mutate A/T in the process of DNA replication and transcription (Schlötterer and Tautz, 1992). In dinucleotide repeats, AC/GT exhibited the highest frequency, which was coincident with the result obtained by Lv et al. (2013). In the development of SSR markers based on transcriptome sequencing in blood clam (*Scapharca kagoshimensis*), AC/GT was also predominant among dinucleotide repeats (Chen et al., 2022). However, the opposite results were found in the Zhikong scallop (*Chlamys farreri*) (Zhan et al., 2008) and bay scallop (*Argopecten irradians*) (Li et al., 2009) from the SSR-enriched library, which showed that the proportion of AG/CT was higher than that of AC/GT in the genome. This difference may be related to the SSR screening method, base composition preference of different coding genes, and methylase activity *in vivo* (Chen et al., 2022).

The location of SSR loci determines their functional roles. SSRs in CDS affect the inactivated or activated genes or protein synthesis process, and SSRs in 3′UTR are involved in transcription slippage or gene silencing, and SSRs in 5′UTR impact gene transcription and translation (Xia et al., 2014; Xu et al., 2020; Liu et al., 2021c). In the present study, 88.92% of microsatellites were located in UTRs, which was much higher than that of CDS regions. One possible reason is that microsatellites located in UTRs are subject to fewer evolutionary constraints and natural selection pressure, thus easily leading to phenotype changes (Xu et al., 2020; Vidya et al., 2021). Moreover, 74.88% of trinucleotide repeats were found to be accumulated in CDSs regions. This might explain that non-trinucleotides negatively selected frameshift mutations, while trinucleotide did not cause frame shift mutation and failed to affect gene expression (Xia et al., 2014; Liu et al., 2021c).

To explore the potential functions of the obtained unigenes, the functional annotation and classification of these unigenes were conducted through BLASTX search in the public databases. GO annotations showed a lot of unigenes distributed to cellular process, metabolic process, cell, cell part, binding, and catalytic activity terms. This suggests that genes encoding these functions may be more conserved across different species and are thus easier to annotate in the database. In addition, KEGG and KOG annotations revealed that many unigenes might participate in the life activities and basal metabolism of *P. trituberculatus* with various biological functions. In summary, these annotation analyses contribute to finding potential genes associated with the growth and development of *P. trituberculatus* for breeding programs. Further studies also should be carried out to identify the molecular functions of these putative genes.

### Population Genetic Diversity

Genetic diversity is the foundational core of ecosystems and species diversity and can reveal population connectivity and adaptive potential of a species as well as provide insight into past events (Fang et al., 2021b; Ma et al., 2021). It is affected by many factors, including artificial selection, genetic drift, migration, and breeding systems and is usually evaluated by the polymorphism information content (*PIC*) and heterozygosity (Zhou, et al., 2019b). Values of *PIC* above 0.5 indicate high polymorphism (Singh et al., 2020). Heterozygosity is an important index to evaluate population variation at the genetic level, and the greater its value, the higher the population genetic diversity (Qin et al., 2013). Li et al. (2011) investigated the genetic diversity of five *P. trituberculatus* populations with eight SSR markers and observed that the mean *H*_*E*_ values ranged from 0.7283 to 0.7704, which revealed a high level of genetic diversity in the wild resources. The current study reports *PIC* values of all SSR loci greater than 0.5, indicating the high polymorphic nature of the loci and their suitability for assessing genetic diversity in the four *P. trituberculatus* populations. The observed and expected heterozygosity values indicated a similar level of genetic diversity among the wild populations, and compared with the wild populations, the genetic diversity of the cultivated population showed a reduction because of lower genetic coefficients. In the estimation of genetic diversity of *P. trituberculatus* populations from Shandong peninsula, Liu et al. (2012) found a similar result. It is possible that genetic decline, genetic drift, and inbreeding result in low genetic variability in farmed stocks (Jorge et al., 2018). Additionally, the domesticated stocks are subjected to artificial selection in a selective breeding program, which may show reduced effective population size, thus leading to a decline in genetic diversity (Wong et al., 2022).

In general, the expected heterozygosity (*H*_*E*_) is more accurate than the observed heterozygosity (*H*_*O*_) for evaluating the level of population genetic diversity because *Ho* is easily influenced by sample sizes (Qin et al., 2013). Based on polymorphic SSR markers, middle (*H*_*E*_ = 0.73–0.84) to high (*H*_*E*_ = 0.916–0.918) genetic diversity of *P. trituberculatus* was revealed by Guo et al. (2013) and Xu and Liu (2011). In this study, the mean *H*_*E*_ ranged from 0.675 to 0.706 which is lower than those observed in the above studies, thus showing a lower level of genetic diversity among the four populations. This may be attributable to the special geographical location of the Bohai Sea. The Bohai Sea is a semi-enclosed shallow water body and has limited connectivity to the Yellow Sea by the Bohai Strait, which restricts the dispersal of crabs and thus results in low genetic diversity (Liu et al., 2012). Moreover, the phenomena of eutrophication and hypoxia, as well as the serious interference from anthropogenic activities such as land-source pollution, aquaculture pollution, and reclamation in the Bohai Sea also reduced the genetic diversity of species (Wang et al., 2021).

### Genetic Differentiation and Variation Among Populations

*F*_*ST*_ is an important gauge of genetic differentiation between populations and is crucial for a better understanding of the genetic relationships. A value of *F*_*ST*_ which falls between 0 and 0.05 shows a low level of genetic differentiation (Wang et al., 2019b). The current study reports the mean *F*_*ST*_ values of 0.021 (*p* < 0.05) which is less than 0.05 (Table 5), indicating low levels of genetic differentiation among the four *P. trituberculatus* populations, which is conformed to the result described by Xu and Liu (2011). Based on the pairwise *F*_*ST*_ ranging from 0.0142 to 0.0498, six SSR loci showed no genetic difference between wild and cultivated populations of *P. trituberculatus* from the Zhejiang coastal region (Li et al., 2015b). This genetic similarity may be accounted for release of hatchery-produced offspring, which results in hybrid germplasm. Liu et al. (2018b) and Liu et al. (2021b) used SSR markers to evaluate the population structure of *P. trituberculatus* in Panjin and Yingkou (Liaoning, China) adjacent to Liaodong Bay, respectively, and found low but significant levels of genetic differentiation (*F*_*ST*_ < 0.05, *p* < 0.05), suggesting that large-scale stock enhancement of *P. trituberculatus* presents potential genetic risks on wild populations, and that the relevant management measures should be formulated to achieve successful stock enhancement and resource restoration for the swimming crab.

During the current study, AMOVA results revealed that total variance within populations (98%) was significantly greater than that among populations (2%). The result corresponded to the genetic variation found in blue swimmer crab (*Portunus pelagicus*) (Chai et al., 2017). Most loci showed a deficit of heterozygotes, which might result from the presence of null alleles, artificial selection, migration, and inbreeding in the population (Guo et al., 2013). Additionally, a majority of SSR loci deviated from the Hardy-Weinberg equilibrium, and this finding might be ascribed to null alleles or a small number of samples. Hence, designing more effective SSR primers to eliminate null alleles, and combining more molecular markers with a larger sample size are essential to elaborate the genetic diversity of *P. trituberculatus* populations in the Bohai Sea. In addition, genetic monitoring is required to preserve the genetic variations for preventing germplasm degradation and making full use of the genetic resources of *P. trituberculatus*.

### Population Genetic Structure

A stable genetic structure is central to species survival. Its disintegration will lead to decreased populations or even extinction. Given the economic significance of *P. trituberculatus*, the understanding genetic structure is crucial for the development of effective management strategies and can provide a genetic tool for breeding and offer a scientific support for resource conservation of this species (Liu et al., 2009). The results of the current study establish that K = 2 is the most likely number of clusters when ΔK is at its highest. This finding confirms that the *P. trituberculatus* specimens from the four populations cluster into two groups including the cultured group and the wild group (Figure 4). Some of the genetic information gained from the cultured samples has been assigned to wild populations, indicating that the ancestral generation of these wild individuals may derive from cultivated populations because of the hatchery-reared seed release activities. In a cultivated group, some genetic information that derives from wild samples can be observed. This observation indicates some degree of introgression of wild populations into the cultivated population, which may be accounted for the fact that fertilized female crabs are caught as broodstocks from the wild to use to artificially culture and produce seeds (Duan et al., 2022b).

The individual-level phylogenetic tree and PCA illuminated that all individuals showed some degree of genetic connectivity, and that the cultured individuals were relatively concentrated in comparison with wild individuals. Despite the annual release activities, the gene exchange between cultivated and wild populations is limited when compared to that between wild populations in the open sea, thus leading to the separation of cultured individuals from all individuals, showing more obvious particularity. Therefore, it is vital to further investigate the genetic structure of wild and cultured populations of *P. trituberculatus* in the Bohai Sea for formulating scientific management measures to prevent mutual interference between them.

## Conclusion

Overall, this study performed assembly of transcriptome sequences, functional annotation, and SSR markers discovery of *P. trituberculatus*. Nineteen polymorphic SSRs were identified and used to investigate the genetic variation and structure of the four *P. trituberculatus* populations from the Bohai Sea. The findings revealed a lower level of genetic diversity in *P. trituberculatus* populations from the Bohai Sea when compared to the other populations from the Yellow Sea and the East China Sea. The pairwise *F*_*ST*_ values showed low but significant genetic differentiation between populations. The population structure analysis, phylogenetic tree, and PCA showed a mixing of wild and cultivated individuals, which corroborated the genetic connectivity between them, but cultivated individuals were distributed more centrally than wild individuals. In addition, heterozygote deficiencies, null alleles, and significant deviation from HWE at many SSR loci were observed. Therefore, practical and effective measures are expected to be taken to reinforce the identification and protection of genetic diversity and prevent degeneration of *P. trituberculatus* germplasm. For example, developing high-quality markers such as SNPs using a chromosome-level genome of *P. trituberculatus* (Tang et al., 2020; Lv et al., 2021), and carrying out a large-scale investigation to fully elucidate the genetic diversity and population structure of *P. trituberculatus* in the Bohai Sea. Additionally, increasing the scale of swimming crab aquaculture, extending the fishing moratorium, and performing long-term genetic monitoring is also helpful for the conservation and utilization of germplasm resources in *P. trituberculatus*. In conclusion, the results improve our understanding of the population genetic structure of *P. trituberculatus* in the Bohai Sea and provide valuable information for the selection breeding of this species.

## Data Availability

The original contributions presented in the study are publicly available. This data can be found in NCBI under accession numbers SUB11453401 and PRJNA836158.
