# Supplementary material for: Development of Microsatellite Markers Based on Transcriptome Sequencing and Evaluation of Genetic Diversity in Swimming Crab (Portunus trituberculatus)
Source: Front Genet. 2022 Jul 18;13:932173. doi: 10.3389/fgene.2022.932173 (PMC9340201; doi:10.3389/fgene.2022.932173)
Supplement: Supplementary file 1 [file DataSheet1.docx]

**Table S1** Sampling information for four *P. trituberculatus* populations.

| Population | Abbreviation | Sample size | Longitude (E°) | Latitude (N°) |
| --- | --- | --- | --- | --- |
| Qinhuangdao | QHD | 60 | 119°60′22″ | 39°95′52″ |
| Huanghua (wild group) | HW | 60 | 117°64′22″ | 38°49′21″ |
| Huanghua (cultured group) | HC | 60 | 117°64′09″ | 38°48′99″ |
| Penglai | PL | 60 | 120°75′66″ | 37°83′29″ |

**Table S2** Frequency of diﬀerent repeat motifs of SSR loci in *P. trituberculatus*.

| Repeats | 5 | 6 | 7 | 8 | 9 | 10 | 11 | 12 | 13 | 14 | 15 | 16 | >16 | Total |
| --- | --- | --- | --- | --- | --- | --- | --- | --- | --- | --- | --- | --- | --- | --- |
| Mono | 0 | 0 | 0 | 0 | 0 | 17762 | 8809 | 5884 | 4243 | 3654 | 3193 | 2625 | 18509 | 64679 |
| Di | 0 | 13019 | 9225 | 7117 | 5695 | 5299 | 14116 | 10120 | 3462 | 3049 | 2589 | 2437 | 34630 | 110758 |
| Tri | 21100 | 13055 | 12068 | 6309 | 1782 | 1500 | 1077 | 826 | 720 | 598 | 525 | 431 | 1932 | 61923 |
| Tetra | 3889 | 1988 | 341 | 239 | 210 | 156 | 109 | 59 | 35 | 35 | 21 | 17 | 55 | 7154 |
| Penta | 758 | 205 | 124 | 64 | 68 | 51 | 22 | 19 | 10 | 4 | 10 | 5 | 3 | 1343 |
| Hexa | 173 | 104 | 56 | 26 | 13 | 9 | 3 | 2 | 0 | 0 | 0 | 0 | 0 | 386 |
| Total | 25920 | 28371 | 21814 | 13755 | 7768 | 24777 | 24136 | 16910 | 8470 | 7340 | 6338 | 5515 | 55129 | 246243 |

Mono: mononucleotide; Di: dinucleotide; Tri: trinucleotide; Tetra: tetranucleotide; Penta: pentanucleotide; Hexa: hexanucleotide.

**Table S3** Frequency distribution of the 13 most frequent SSR repeat motifs in *P. trituberculatus*.

| Repeats | 5 | 6 | 7 | 8 | 9 | 10 | 11 | 12 | 13 | 14 | 15 | 16 | >16 | Total |
| --- | --- | --- | --- | --- | --- | --- | --- | --- | --- | --- | --- | --- | --- | --- |
| A/T | 0 | 0 | 0 | 0 | 0 | 16961 | 8413 | 5674 | 4084 | 3612 | 3165 | 2609 | 18128 | 62646 |
| AC/GT | 0 | 7081 | 5237 | 3904 | 3161 | 2850 | 7418 | 5181 | 1875 | 1564 | 1333 | 1187 | 16203 | 56994 |
| AG/CT | 0 | 4235 | 3155 | 2564 | 2055 | 2105 | 6159 | 4722 | 1476 | 1381 | 1163 | 1185 | 17287 | 47487 |
| ACC/GGT | 6257 | 3968 | 2916 | 1650 | 446 | 342 | 182 | 113 | 104 | 57 | 69 | 23 | 97 | 16224 |
| AGG/CCT | 4217 | 2677 | 2415 | 1108 | 454 | 356 | 323 | 249 | 176 | 155 | 173 | 106 | 305 | 12714 |
| AAT/ATT | 1940 | 1193 | 2082 | 1068 | 182 | 232 | 159 | 139 | 173 | 111 | 110 | 117 | 535 | 8041 |
| AGC/CTG | 2938 | 1476 | 836 | 742 | 208 | 138 | 108 | 54 | 48 | 54 | 24 | 40 | 65 | 6731 |
| ACT/AGT | 1407 | 952 | 1977 | 673 | 175 | 142 | 124 | 105 | 86 | 94 | 61 | 59 | 573 | 6428 |
| AT/AT | 0 | 1597 | 765 | 621 | 463 | 334 | 536 | 217 | 111 | 104 | 93 | 65 | 1140 | 6046 |
| AAG/CTT | 1353 | 796 | 691 | 369 | 100 | 103 | 93 | 59 | 51 | 47 | 38 | 43 | 204 | 3947 |
| AAC/GTT | 1084 | 890 | 610 | 339 | 111 | 120 | 49 | 71 | 52 | 64 | 34 | 34 | 110 | 3568 |
| ATC/ATG | 1006 | 642 | 410 | 275 | 85 | 51 | 28 | 35 | 27 | 14 | 14 | 8 | 41 | 2636 |
| C/G | 0 | 0 | 0 | 0 | 0 | 801 | 396 | 210 | 159 | 42 | 28 | 16 | 381 | 2033 |


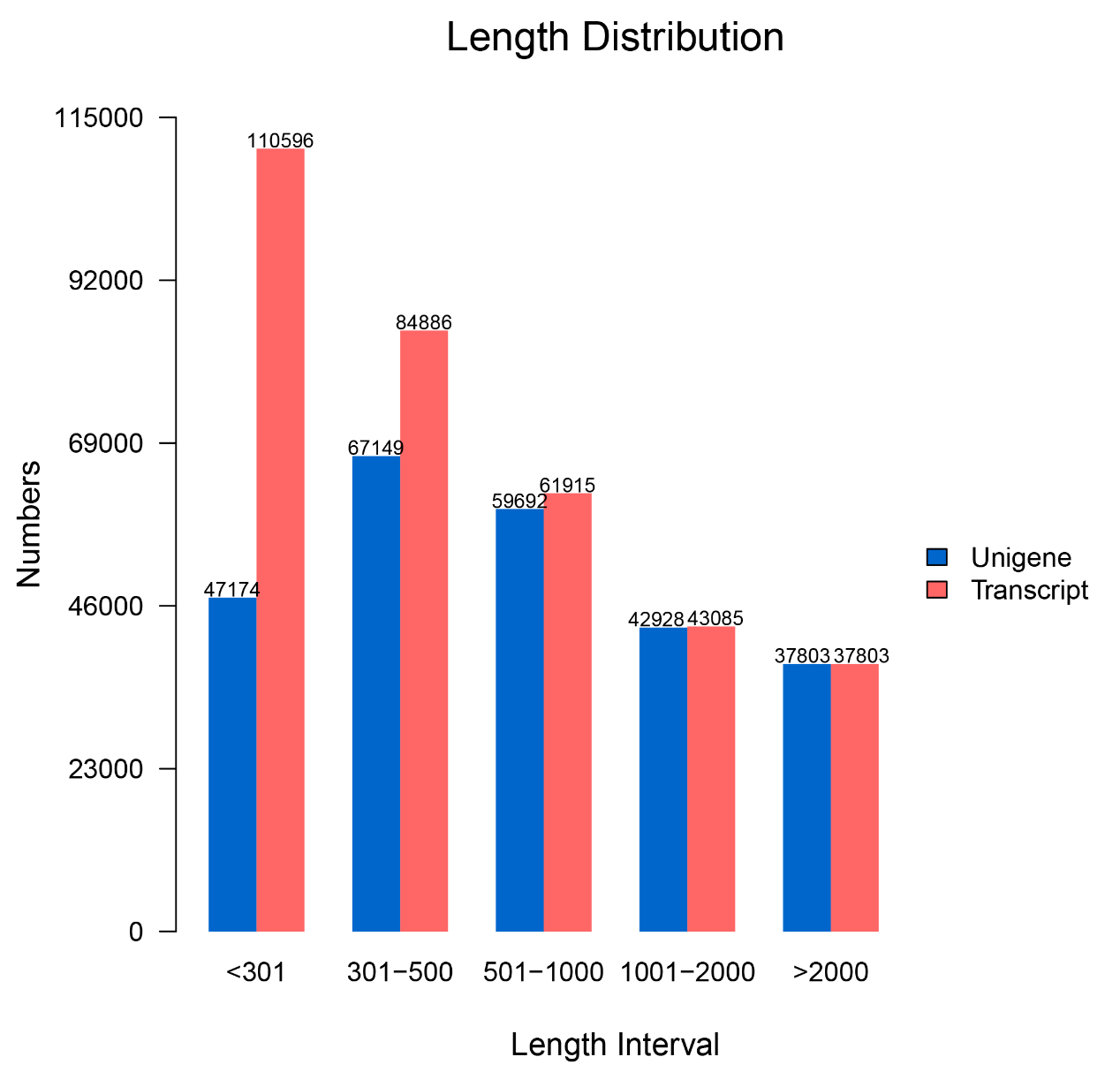


**Figure S1** Length distribution of unigenes and transcripts of *P. trituberculatus*.


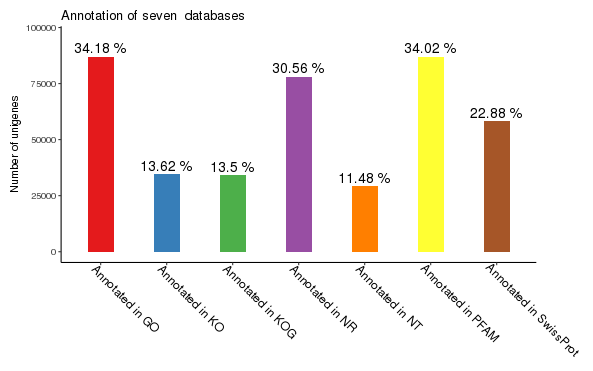


**Figure S2** Annotation of seven databases of *P. trituberculatus*.


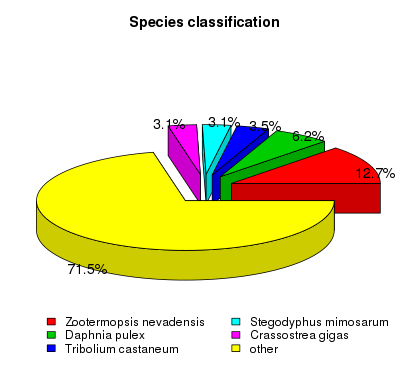


**Figure S3** The species distribution of Nr annotations.


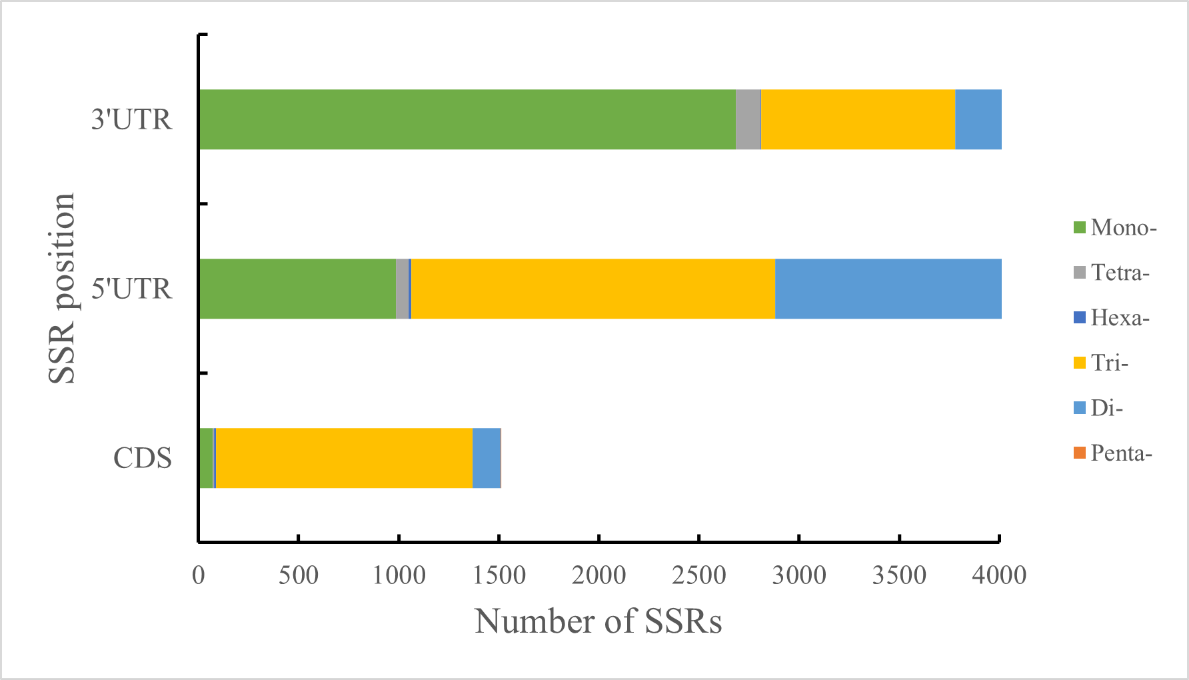


**Figure S4** Distribution of six repeat motifs in different unigene positions.


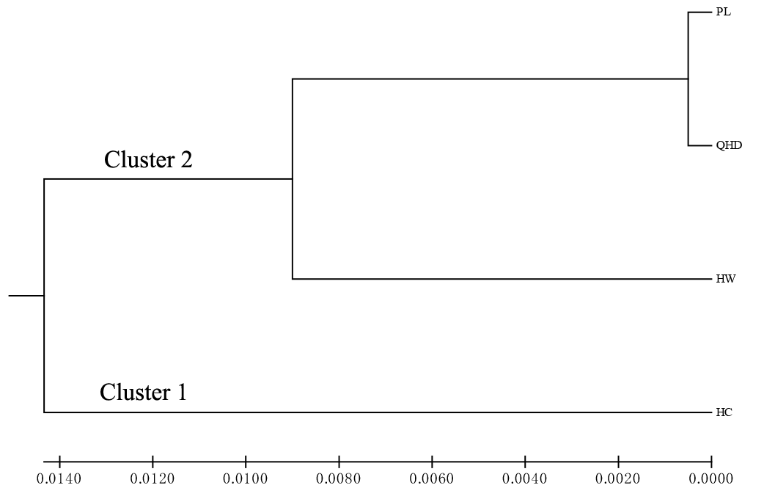


**Figure S5** Phylogenetic tree among the four different populations of *P. trituberculatus*.
